# Frameshift mutations in coding repeats of protein tyrosine phosphatase genes in colorectal tumors with microsatellite instability

**DOI:** 10.1186/1471-2407-8-329

**Published:** 2008-11-10

**Authors:** Sebastian Korff, Stefan M Woerner, Yan P Yuan, Peer Bork, Magnus von Knebel Doeberitz, Johannes Gebert

**Affiliations:** 1 Department of Applied Tumor Biology, Institute of Pathology, University of Heidelberg, Heidelberg, Germany; 2 European Molecular Biology Laboratory, Heidelberg, Germany; 3 Max Delbrueck-Centrum for Molecular Medicine, Berlin, Germany

## Abstract

**Background:**

Protein tyrosine phosphatases (PTPs) like their antagonizing protein tyrosine kinases are key regulators of signal transduction thereby assuring normal control of cellular growth and differentiation. Increasing evidence suggests that mutations in PTP genes are associated with human malignancies. For example, mutational analysis of the tyrosine phosphatase (PTP) gene superfamily uncovered genetic alterations in about 26% of colorectal tumors. Since in these studies tumors have not been stratified according to genetic instability status we hypothesized that colorectal tumors characterized by high-level of microsatellite instability (MSI-H) might show an increased frequency of frameshift mutations in those PTP genes that harbor long mononucleotide repeats in their coding region (cMNR).

**Results:**

Using bioinformatic analysis we identified 16 PTP candidate genes with long cMNRs that were examined for genetic alterations in 19 MSI-H colon cell lines, 54 MSI-H colorectal cancers, and 17 MSI-H colorectal adenomas. Frameshift mutations were identified only in 6 PTP genes, of which PTPN21 show the highest mutation frequency at all in MSI-H tumors (17%).

**Conclusion:**

Although about 32% of MSI-H tumors showed at least one affected PTP gene, and cMNR mutation rates in PTPN21, PTPRS, and PTPN5 are higher than the mean mutation frequency of MNRs of the same length, mutations within PTP genes do not seem to play a common role in MSI tumorigenesis, since no cMNR mutation frequency reached statistical significance and therefore, failed prediction as a Positive Selective Target Gene.

## Background

Chromosomal instability (CIN) and aneuploidy are molecular features of most sporadic colorectal cancers (~85%) and may confer a worse prognosis [1-3]. About 15% of colorectal cancers (CRC) show microsatellite instability (MSI) due to defective DNA mismatch repair (MMR; [4]). In hereditary non-polyposis colorectal cancer (HNPCC/Lynch syndrome; about 5% of all CRC cases) most of the tumors display this MSI phenotype [5]. As a common molecular theme, MMR-deficient MSI tumors of the colon and other organs accumulate numerous insertion/deletion mutations [6,7] not only at non-coding but also at coding microsatellites (cMS) that cause translational frameshifts and abrogate normal protein function. Such frameshift protein derived *neo*-peptides can be highly immunogenic and are capable to induce cytotoxic T-cell-mediated killing of MSI-H tumor cells in vitro [8-11]. Frameshift mutations in cMS sequences of a large number of candidate genes have been identified [12-16] and mutations in some of them (TGFBR2, ACVR2, BAX; TCF-4) appear to provide a growth advantage to affected cells [17-20]. Both, sporadic and HNPCC-associated colorectal MSI-H cancers, show distinct clinico-pathological characteristics that include frequent proximal site, diploidy, poor differentiation, less distant metastases, peritumoral lymphocytic infiltrate, comparably good prognosis, and altered chemoresponsiveness [6,7,21-26]. Increasing evidence suggests that cMS mutations in a limited number of target genes appear to be selected for during MSI carcinogenesis and might account for some of these clinico-histopathological features.

Protein tyrosine phosphatases (PTPs) like their antagonizing protein tyrosine kinases are key regulators of signal transduction thereby assuring normal control of cellular growth and differentiation [27]. Alterations in the delicate balance between tyrosine phosphorylation and dephosphorylation contribute to the pathogenesis of different inherited or acquired human diseases including autoimmunity, diabetes, and cancer [27-29]. Several studies indicate that mutations in PTP genes may be involved in colorectal carcinogenesis. For example, increased *PTPRA *mRNA levels have been observed in late stage colorectal tumors [30] and frequent overexpression of the human transmembrane-type *PTP SAP-1 *may occur relatively late in the adenoma-carcinoma sequence [31]. Expression profiling studies also suggested that PTPs appear to be involved in metastasis of colorectal cancer [32]. In a similar approach, differential expression of the human *PTPN21 *gene was observed when comparing MSI-H with microsatellite stable (MSS) colorectal cancer cell lines [33] and mutations in this gene were reported to occur in a subset of MSI-H colorectal carcinomas [34]. Additionally, a somatic mutation in the non-receptor PTP Shp2, encoded by the *PTPN11 *gene, has been detected in a single colon tumor with an increased frequency of somatic alterations, but without microsatellite instability [35]. Moreover, identification of the murine PTP gene Ptprj as a modifier locus conferring susceptibility to colorectal cancer also led to the detection of frequent deletions of the human *PTPRJ *gene in primary colon cancers [36]. Finally, systematic mutational analysis of the human PTP gene super family identified somatic mutations in six PTPs (*PTPRF*, *PTPRG*, *PTPRT*, *PTPN3*, *PTPN13*, *PTPN14*), affecting 26% of colorectal cancers [37].

However, whether coding mononucleotide repeats (cMNR) in PTP genes are specific targets of frameshift mutations in MMR-deficient colorectal tumors is still unknown. In the present study we identified 16 human PTP genes harboring coding region microsatellites and determined their mutation frequencies in MSI-H colorectal tumors. About 32% of MSI-H tumors showed frameshift mutations in any of these PTP genes. However, gene-specific cMNR mutation frequencies did not reach statistical significance according to our recently proposed model for Selective Target Gene prediction [16]. Hence, there is no significant statistical support for a common involvement of any of these PTP genes in MSI colon carcinogenesis.

## Methods

### Cell Lines and Tumor Tissues

Most of the cell lines analyzed in the present study have been described previously [15]. Additional human colorectal cancer cell lines were obtained from Cell Line Services (CLS), Heidelberg, Germany (Colo94H, Colo205, HCT8, SW1116, SW403, T84) or kindly provided by Dr. M. Brattain, University of Texas, Health Science Center, San Antonio, TX (CBS, FET), Dr. J. Wilson, Case Western Reserve University, Cleveland, Ohio (Vaco5, Vaco6, Vaco432, Vaco457), or Dr. I. Fidler (KM12). Cells were grown in RPMI supplemented with 10% fetal calf serum (Life Technologies, Karlsruhe, Germany). Formalin-fixed and Paraffin-embedded tumor and matched normal mucosae samples were treated as described [38]. The MSI status of tumor cell lines (19 MSI-H, 17 MSS), primary colorectal tumors (54 MSI-H and matched normal mucosae), and colorectal adenomas (17 MSI-H, 6 MSS, and matched normal mucosae) has been determined using the NCI/ICG-HNPCC microsatellite marker panel [39] as previously described [38]. Whole blood DNA samples of 60 healthy donors served as additional MSS controls. Informed consent was obtained from all patients and blood donors.

### Candidate cMNR Sequences in PTP Genes

Human PTP genes were sought in our cMNR database ([15] accessible at , MNR_ensembl) that covers all human candidate coding mononucleotide repeats in the entire human genome annotated in the Ensembl database (Ensembl 19.34b, 2004). Candidate cMNRs with repeat length of at least 7 repeat units were considered for further analysis.

### cMNR Instability Analysis

Primer design, DNA fragment and data analysis was performed as described [40]. Primer sequences and annealing temperatures are shown in Table 1 [see Additional file 1]. Frameshift mutations were verified by DNA sequence analysis.

## Results

### cMNR instability of PTPs in MSI-H colorectal cancer cell lines

**Figure 1:**
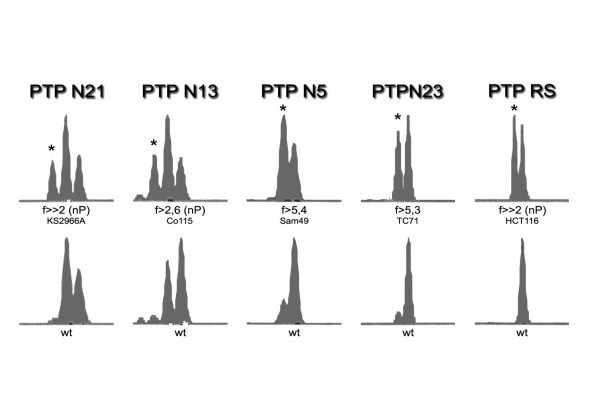
**Electropherograms of allelic shifts**. Electropherograms of shifted alleles. DNA fragment analysis of a representative set of PTP cMNRs in a MSI-H colorectal tumor (KS2966A, Sam49) and MSI-H cancer cell lines (Co115, TC71, and HCT116). Corresponding wildtype alleles (wt, lower panel) are indicated together with tumor cell-specific allelic shifts marked by asterisks (upper panel). Allele intensities were determined (peak area) and ratios (f) of wildtype and novel alleles in primary tumors and cell lines were calculated, defining a 2-fold difference as threshold for allelic shifts.

A candidate set of 16 PTP genes that contained cMNR sequences composed of at least 7 repeat units were retrieved from our cMNR database (Table 1 [see Additional file 1], MNR_ensembl is accessible at ). Using DNA fragment analysis we examined cMNR length alterations of these candidate genes in 19 MSI-H and 17 MSS colorectal cancer cell lines. cMNR frameshift mutations occurred exclusively in MSI-H colorectal cancer cell lines and affected four PTP genes (Tables 2 and 4 [see Additional file 1]; *PTPN21*, *PTPN13*, *PTPRS*, *PTPN23*). Results of fragment analysis are exemplarily shown in figure 1. The highest mutation frequencies were associated with the A_8 _cMNRs in *PTPN13 *(22%) and *PTPN21 *(26%). All mutations occurred in a heterozygous state and predominantly represented single nucleotide deletions (minus 1 allele, n = 10). Two were single nucleotide insertions (plus 1 allele, *PTPN13 *and *PTPN21*). No larger shifts could be detected. Overall, the larger fraction of MSI-H colorectal cancer cell lines (63%; 12/19) showed frameshift mutations in any of the four affected PTP genes.

### PTP cMNR Mutations in MSI-H Primary Colorectal Carcinomas

**Figure 2:**
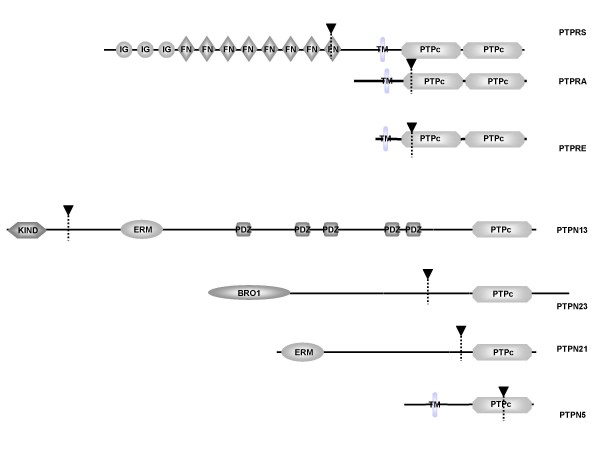
**Domain structure of selected PTPs and sites of cMNR frameshift mutations**. Structure of PTP proteins. Tyrosine phosphatase domains (PTPc) as well as frameshift mutation sites (arrowhead, stippled lines) are indicated. Remaining domain abbreviations: IG immunoglobulin like domain, FN fibronectin type 3 domain, KIND kinase non-catalytic C-lobe domain, ERM ezrin/radixin/moesin domain, PDZ Domain present in PSD-95, Dlg, and ZO-1/2., BRO1 BRO1-like domain, TM transmembrane domain. Despite PTPN5 is a non-receptor phosphatase there is a TM domain annotated within Ensembl.

We next analyzed the same set of 16 PTP genes for cMNR frameshift mutations in 54 MSI-H colorectal carcinomas and adjacent normal mucosa. Frameshift mutations in these primary tumors were identified in 6 PTP genes (*PTPN21*, *PTPRS*, *PTPN5*, *PTPN23*, *PTPRA*, *PTPRE*) comprising mutation targets shared by (*PTPN21*, *PTPRS*, *PTPN23*) or different from (*PTPN5*, *PTPRA*, *PTPRE*) those in the MSI-H cell lines (Tables 3 and 4 [see Additional file 1], Figure 1). In MSS mucosa samples such changes almost never occurred (1/703 analyses). Two genes, *PTPN21 *and *PTPRS*, were most frequently affected in primary colorectal tumors (16% and 12%, respectively). However, primary tumors lacked frameshift mutations in the A_8 _repeat of *PTPN13 *and thus appeared to arise only in cultured MSI-H CRC cell lines. Since LS174T and LS180 are derived from the same primary tumor [41,42] and the overall mutation frequency is quite low it is very likely that this mutation already manifested within the primary tumor. Vice versa, mutations in three other PTPs (*PTPN5*, *PTPRA*, *PTPRE*) remained restricted to primary tumors and occurred at very low frequency (Tables 3 and 4 [see Additional file 1]). Overall, frameshift mutations in any of these 6 PTP genes were observed in 32% of MSI-H tumors. The most common type of mutation were single nucleotide deletions (16/21, 76%) and all frameshift mutations appeared to affect only one allele (heterozygous). Interestingly, all PTP mutations are located within or upstream of the phosphatase domains and hence are expected to impair or completely abrogate phosphatase activity (Figure 2).

### PTPN21 cMNR Mutations in MSI-H Colorectal Adenomas

Since mutations in the A_8 _coding repeat of *PTPN21 *were the most frequent genetic alterations shared by MSI-H cancer cell lines and primary tumors we also analyzed this coding repeat in 17 MSI-H and 6 MSS colorectal adenoma samples. As expected, no cMNR frameshift mutations were detected in the MSS adenomas. However, in 2 of 17 MSI-H adenomas we identified deletion and insertion mutations (Table 4 [see Additional file 1], Figure 1). These results rather argue for an early event during MSI colorectal carcinogenesis.

## Discussion

The goal of this study was to determine type, frequency, and pattern of cMNR frameshift mutations in protein PTP genes in MSI-H colorectal cancer cell lines and primary colon tumors. This gene family was selected because PTPs are expected to exert tumor suppressive function and thus represent potential targets of inactivating mutations in DNA mismatch repair deficient tumors. Among the large number of PTP genes in the human genome we selected 16 PTP genes that contained coding region microsatellites of increased length (≥ 7 repetitive units). Our results provide evidence for frameshift mutations in six of these PTP genes (*PTPN21*, *PTPRS*, *PTPN5*, *PTPN23*, *PTPRA*, *PTPRE*) affecting about 32% of MSI-H colorectal cancers. This frequency differs slightly from a previous study that reported PTP mutations in 26% of unselected colorectal carcinomas [37]. Although most of the coding repeat-harboring PTP genes (13/16 candidates) analyzed by us have also been examined by Wang and co-workers, different sets of mutated PTP genes were identified in both studies. In particular, we observed tumor-specific genetic alterations in *PTPN21*, *PTPN23*, *PTPN5*, *PTPRA*, *PTPRE *and *PTPRS *whereas mutations reported by Wang et al. were confined to *PTPRF*, *PTPRG*, *PTPRT*, *PTPN3*, *PTPN13*, and *PTPN14*. This difference most probably can be explained by the different screening strategies used in these two studies. Wang et al. performed their initial mutational pre-screening on a set of 18 unselected colorectal tumors, This subset would be expected to include only 2 to 3 MSI tumors based on a general MSI frequency of 15% reported for unselected colorectal cancers. Since the type of mutation and the spectrum of affected genes is remarkably different among MSS and MSI-H tumors, the observed differences are not unexpected. However, there were also some consistent findings in both studies. For example, PTPN13 was examined by both groups but no cMNR mutations were observed in a cumulative number of 69 MSI-H CRC samples ([43] and data presented here). Interestingly, Wang et al. described two nonsense or frameshift mutations at the expected MNR position within *PTPN13*. Unfortunately, any information about the MSI status of these tumors is missing. So, our investigation represent a reasonable completion of a systematic analysis of human PTP genes in human colorectal cancers.

Recently, we proposed a statistical model that allows to predict positively or negatively selected target genes of MSI tumorigenesis in an organ-specific manner based on cMNR mutation frequencies [16]. According to the actual release of this model (, release 200711), cMNRs of 7 or 8 repetitive units – in the absence of any biological selection pressure – are expected to show a mean somatic mutation rate of about 4% and 9% respectively. In order to qualify as positively selected MSI target genes, tumor-specific mutation frequencies for cMNRs of this length have to exceed 22% and 28%, respectively. The three most frequently mutated PTPs identified in the present study (*PTPN21 *[A_8_], *PTPRS *[C_7_], *PTPN5 *[C_7_]) show mutation frequencies of 17%, 12%, and 6%, respectively. The final overall mutation frequency of *PTPN21 *A_8 _(14.4%) is even slightly lower, when considering data from the literature [14]. Certainly, we did not extend our mutation search to the entire coding sequence of each PTP gene, and therefore, additional mutations outside the cMNR sequence cannot be excluded. Furthermore, detailed studies of individual tumor types have provided compelling evidence that mutations of different genes but within the same pathway can have similar functional effects, i.e. leading to its disruption and providing a growth advantage to affected cells [37], as it has been proposed for apoptotic genes like *Fas*, *Apaf-1*, and *Bcl-10 *[44]. In conclusion, although these gene-specific mutation rates are higher than the mean mutation frequencies for this length of repeats they do not reach statistical significance and prediction about positive Selective Target Genes in MSI-H tumorigenesis is not feasible. Therefore, human PTP genes do not seem to play a common role in MSI-H tumorigenesis. However, contribution to individual tumor development cannot be excluded. Furthermore, the observation of 2 mutations within a set of 17 MSI-H adenomas in *PTPN21 *argues for an early event in malignant transformation.

Despite some structural diversity, the six PTP proteins found to be mutated in the present study share a catalytically active phosphatase that resides in one (*PTPN21*, *PTPN23*, *PTPN5*, *PTPRS*) or two (*PTPRA*) C-terminal domains [45]. Notably, all cMNR frameshift mutation sites are located upstream or within the first half of these catalytic domains (see Figure 2) resulting in truncated proteins that are expected to show partial or complete loss of phosphatase activity. However, all PTP frameshift mutations only affected a single allele leaving the cMNR on the remaining allele intact. The presence of contaminating normal inflammatory cells in these tumors may well account for this observation. Alternatively, the remaining wildtype copy may be silenced by epigenetic mechanisms. The absence of biallelic mutations in the analyzed tumors could be due to a dominant negative fashion or affection of gene dosage [46,47]. Shortened transcripts of murine PTPs lacking catalytic or interaction domains by alternative splicing can act in a dominant negative manner [48].

From our analysis of preneoplastic lesions we further conclude that *PTPN21 *frameshift mutations also occur in MSI-H colorectal adenomas albeit at lower frequency (12%) thereby indicating an early step during MSI tumorigenesis. At the protein level, *PTPN21 *is known to bind to and activate c-Src and Etk protein kinases [49,50]. Interestingly, activated Etk has been reported to trigger apoptosis in breast cancer cells via Stat1 and p21 [51]. By analogy, we hypothesize that inactivation of *PTPN21 *by cMNR frameshift mutations in MSI-H colon cancer cells might lead to Etk inactivation and subsequent inhibition of apoptosis.

## Conclusion

The work presented here shows a systematic investigation using an evidence based approach: cMNRs of human PTP genes with the highest chance for mutational events in MSI-H colorectal tumors were selected. Although we observed a similar overall mutation frequency in MSI colorectal cancers compared to the results reported by Wang et al. for unselected colorectal cancers, a different set of PTP genes appears to be mutated in MSI tumors. Human PTP genes identified in the present study seem to play no common role in the tumorigenesis of MSI-H tumors, supported by statistical considerations. However, within individual MSI-H carcinogenesis such mutations could have had impact to malignant transformation, but so far no functional data exist that could confirm this hypothesis.

## Competing interests

The authors declare that they have no competing interests.

## Authors' contributions

SK carried out the molecular analyses, participated in the data analysis and interpretation, and drafted the manuscript, SMW participated in the design of the study, in the data analysis and interpretation, performed the statistical analysis, and contributed to manuscript writing, YPY and PB did the bioinformatic analysis and established the human cMNR database and the list of candidate genes, MvKD provided project supervision, JG provided the general concept, design of study, supervision and contributed to manuscript writing.

All authors read and approved the final manuscript.

## Note

**Table 1**: **Coding microsatellites in candidate PTP genes examined in this study**.

For each of the 16 PTPs is given the official HUGO identification (hugoID), the actual Ensembl entry ID at  (ENSG), the chromosomal localization (Chr.), the former EMBL accession number (Acc. no.), the type of tract (nucleotide and length, cMNR), the exact position of the tract in relation to the sequence of the EMBL entry (Pos.), the annealing temperature for PCR (Ta) as well as the primer sequences (5' -> 3', sense and antisense) used for fragment analysis of the respective tract.

**Table 2**: **Allelic cMNR mutation status in MSI-H colorectal cancer cell lines**.

Wild type: wt; Deletions of one or two mononucleotides: -1, -2; Insertions of one or two mononucleotides: +1, +2; Analysis failed or not evaluable: 0.

**Table 3**: **Allelic cMNR mutation status in MSI-H colorectal carcinomas**.

For abbreviations see legend of Table 2.

**Table 4**: PTP cMNR mutation frequencies in MSI-H colorectal cancer cell lines, tumors, and adenomas*.

*Analysis restricted to *PTPN21*.

## Pre-publication history

The pre-publication history for this paper can be accessed here:



## Supplementary Material

Additional file 1 **Supplemental material and results information.** Table 1 shows the PTP candidate list including primer systems used for the fragment analysis, Tables 2 and 3 present cMNR mutation status information in MSI-H colorectal cancer cell lines and tumors, and Table 4 summarizes the mutation frequencies of the PTP candidate genes in MSI-H colorectal cancer cell lines, tumors, and adenomas. Click here for file
