# Supplementary material for: Frameshift mutations in coding repeats of protein tyrosine phosphatase genes in colorectal tumors with microsatellite instability
Source: BMC Cancer. 2008 Nov 10;8:329. doi: 10.1186/1471-2407-8-329 (PMC2586028; doi:10.1186/1471-2407-8-329)
Supplement: Additional file 1 — Supplemental material and results information. Table 1 shows the PTP candidate list including primer systems used for the fragment analysis, Tables 2 and 3 present cMNR mutation status information in MSI-H colorectal cancer cell lines and tumors, and Table 4 summarizes the mutation frequencies of the PTP candidate genes in MSI-H colorectal cancer cell lines, tumors, and adenomas. [file 1471-2407-8-329-S1.doc]

**Table 1**

| **hugoID** | **ENSG** | **Chr.** | **Acc.no.** | **cMNR** | **Pos.** | **Ta** | **sense** | **antisense** |
| --- | --- | --- | --- | --- | --- | --- | --- | --- |
| ***PPFIA1*** | ENSG00000131626 | 11 | D49354 | A7 | 545 | 62 | CAG GAT GCC TTG GGA CTT AG | GCT TAG GCA CAA CTG AAA ACC |
| ***PPFIA2*** | ENSG00000139220 | 12 | AF034799 | A7 | 3334 | 59 | GGA ATG CTT GGT AGA TGC AA | TCA TTT TAA TTA TTC TTT GGG GAA A |
| ***PTPLA*** | ENSG00000165996 | 10 | AF114494 | T7 | 660 | 60 | GCA ATA GAC CTG CAA TGT AAA GTC | TTC ACC AGC AAC TCC AAC AG |
| ***PTPN12*** | ENSG00000127947 | 7 | M93425 | A7 | 2158 | 62 | CGG AAT GGA GTG AAC TTC AAA | CAG CTA TTT CAA CAA CTG CAA GA |
| ***PTPN13*** | ENSG00000163629 | 4 | D21209 | A8 | 911 | 60 | CAT TCT CCC CTT ACC AGT TCA | CAA AGC AAG TCC ATG GAT GA |
| ***PTPN21*** | ENSG00000070778 | 14 | X79510 | A8 | 2870 | 60 | TTT CAC AGG GGT TGT ATT TGT G | AGG GCA GCC AGT TTA AGA GG |
| ***PTPN23*** | ENSG00000076201 | 3 | AL110210 | C7 | 1097 | 59 | AGG ACT CCT ACC CCC ACA AT | GCT GGC AGA GGG TCT TGA |
| ***PTPN4*** | ENSG00000088179 | 2 | M68941 | T7 | 1728 | 60 | TTC TTC CGT TTG GAC AGA CC | TCC AAC AAT AAA GCC TTA ACA CA |
| ***PTPN5*** | ENSG00000110786 | 11 | U27831 | C7 | 1319 | 62 | GAG GCC TGA AGC ATT ACT GG | ACC TGC AGT GGA CGA TGA T |
| ***PTPRA*** | ENSG00000132670 | 20 | X54130 | A7 | 803 | 62 | TGT TTA TCC CTG CTA ATA ATT CCT C | AAG ACA CTC ACA AGG CAA GAT G |
| ***PTPRB*** | ENSG00000127329 | 12 | X54131 | T7 | 4840 | 60 | TTT GAT GAG GAC CTG AAG GAA | GGT GAC ATG GCA GTT GGT AG |
| ***PTPRE*** | ENSG00000132334 | 10 | X54134 | A7 | 533 | 62 | CAC CAT TGA TCA TGC AAA ACA | GTC ATT GCC ATC TGG ACA CA |
| ***PTPRH*** | ENSG00000080031 | 19 | D15049 | A7 | 1420 | 58 | CCC GGA ACC TTG TAC ACA TT | CTC ACC AAA ACA GGG AGT GG |
| ***PTPRR*** | ENSG00000153233 | 12 | U42361 | A7 | 1661 | 60 | ATG GTT TGG CAG GAA GAC AG | TCC ATG GCA ATT TTA CTA ACA CA |
| ***PTPRS*** | ENSG00000105426 | 19 | U35234 | C7 | 3174 | 63 | ACG GCC TAT GAC CTC CAA GT | GAC CAC GAG GAA GCA GAA AA |
| ***PTPRZ1*** | ENSG00000106278 | 7 | M93426 | A7 | 284 | 59 | TTT TGG GCT ATT ACA TGT TGG A | TGT AAA GTT TAA TTT TTG CAT GGT |

# Table 2

|  | *PPFIA1* | *PPFIA2* | *PTPLA* | *PTPN12* | *PTPN13* | *PTPN21* | *PTPN23* | *PTPN4* | *PTPN5* | *PTPRA* | *PTPRB* | *PTPRE* | *PTPRH* | *PTPRR* | *PTPRS* | *PTPRZ1* |
| --- | --- | --- | --- | --- | --- | --- | --- | --- | --- | --- | --- | --- | --- | --- | --- | --- |
| Co115 | wt | 0 | wt | wt | –1wt | wt | wt | wt | wt | wt | wt | wt | wt | wt | wt | wt |
| Colo60H | wt | 0 | wt | wt | wt | wt | wt | wt | wt | wt | wt | wt | wt | wt | –1wt | wt |
| HCT116 | wt | 0 | wt | wt | wt | wt | wt | wt | wt | wt | wt | wt | wt | wt | –1wt | wt |
| HDC108 | wt | 0 | wt | wt | wt | wt | wt | wt | wt | wt | wt | wt | wt | wt | wt | 0 |
| HDC143 | wt | 0 | wt | wt | wt | wt+1 | wt | wt | wt | wt | wt | wt | wt | wt | wt | 0 |
| HDC9 | wt | 0 | 0 | wt | wt | wt | wt | wt | wt | wt | wt | wt | wt | wt | wt | 0 |
| HRT18 | wt | 0 | wt | wt | wt | wt | wt | wt | wt | wt | wt | wt | wt | wt | wt | wt |
| KM12 | wt | 0 | wt | wt | wt | –1wt | wt | wt | wt | wt | wt | wt | wt | wt | wt | wt |
| LoVo | wt | wt | wt | wt | 0 | wt | wt | wt | wt | wt | wt | wt | wt | wt | wt | wt |
| LS174T | wt | wt | 0 | wt | –1wt | wt | wt | wt | wt | wt | wt | wt | wt | wt | wt | wt |
| LS180 | wt | 0 | wt | wt | –1wt | wt | wt | wt | wt | wt | wt | wt | wt | wt | wt | wt |
| RKO | wt | 0 | wt | wt | wt | –1wt | wt | wt | wt | wt | wt | wt | wt | wt | wt | wt |
| SW48 | wt | wt | wt | wt | wt | wt | wt | wt | wt | wt | wt | wt | wt | wt | wt | wt |
| TC7 | wt | 0 | wt | wt | wt | wt | wt | wt | wt | wt | wt | wt | wt | wt | wt | wt |
| TC71 | wt | wt | wt | wt | wt | wt | –1wt | wt | wt | wt | wt | wt | wt | wt | wt | wt |
| VaCo432 | wt | 0 | wt | wt | wt | wt | wt | wt | wt | wt | wt | wt | wt | wt | wt | wt |
| VaCo457 | wt | 0 | wt | wt | wt+1 | wt | wt | wt | wt | wt | wt | wt | wt | wt | wt | wt |
| VaCo5 | wt | 0 | wt | wt | wt | –1wt | wt | wt | wt | wt | wt | wt | wt | wt | wt | wt |
| VaCo6 | wt | 0 | wt | wt | wt | –1wt | wt | wt | wt | wt | wt | wt | wt | wt | wt | wt |

# Table 3

| **sample** | **PPFIA1** | **PPFIA2** | **PTPLA** | **PTPN12** | **PTPN13** | **PTPN21** | **PTPN23** | **PTPN4** | **PTPN5** | **PTPRA** | **PTPRB** | **PTPRE** | **PTPRH** | **PTPRR** | **PTPRS** | **PTPRZ1** |
| --- | --- | --- | --- | --- | --- | --- | --- | --- | --- | --- | --- | --- | --- | --- | --- | --- |
| **sam01** | wt | wt | wt | wt | wt | wt | wt | wt | wt | wt | wt | wt | wt | wt | wt | wt |
| **sam02** | wt | wt | wt | wt | wt | wt | wt | wt | wt | wt | wt | wt | wt | wt | –1wt | wt |
| **sam03** | wt | 0 | wt | wt | wt | –1wt | wt | wt | wt | wt | wt | wt | wt | wt | wt | wt |
| **sam04** | wt | wt | wt | wt | wt | wt | wt | wt | wt | wt | wt | wt | wt | wt | wt | wt |
| **sam05** | wt | wt | wt | wt | wt | wt | wt | wt | wt | wt | wt | wt | wt | wt | wt | wt |
| **sam06** | wt | wt | wt | wt | wt | wt | wt | wt | wt | wt | wt | wt | wt | wt | wt | wt |
| **sam07** | wt | wt | wt | wt | wt | wt | wt | wt | wt | wt | wt | wt | wt | wt | wt | wt |
| **sam08** | wt | wt | wt | wt | wt | wt | wt | wt | wt | wt | wt | wt | wt | wt | wt | wt |
| **sam09** | wt | wt | wt | wt | wt | wt | wt | wt | wt | wt | wt | wt | wt | wt | wt | wt |
| **sam10** | wt | wt | wt | wt | wt | wt | wt | wt | wt | wt | wt | wt | wt | wt | wt+1 | wt |
| **sam11** | wt | wt | wt | wt | wt | wt | wt | wt | wt | wt | wt | wt | wt | wt | wt | wt |
| **sam12** | wt | wt | wt | wt | wt | wt | wt | wt | wt | wt | wt | wt | wt | wt | wt | wt |
| **sam13** | wt | 0 | wt | wt | 0 | wt | wt | 0 | wt | 0 | wt | wt | wt | wt | wt | wt |
| **sam14** | wt | 0 | wt | wt | 0 | wt | wt | 0 | wt | 0 | 0 | wt | wt | wt | wt | wt |
| **sam15** | 0 | 0 | 0 | 0 | 0 | wt | 0 | 0 | wt | 0 | 0 | 0 | wt | 0 | wt | wt |
| **sam16** | wt | wt | wt | wt | wt | wt | wt | wt | wt+1 | wt | wt | wt | wt | wt | wt | wt |
| **sam17** | wt | wt | wt | wt | wt | wt | wt | wt | wt | wt | wt | wt | wt | wt | wt | wt |
| **sam18** | wt | 0 | 0 | wt | 0 | wt | wt | 0 | wt | 0 | 0 | wt | wt | wt | wt | wt |
| **sam19** | wt | 0 | wt | wt | 0 | wt | wt | 0 | wt | 0 | wt | wt | wt | wt | wt | wt |
| **sam20** | wt | wt | wt | wt | wt | wt | wt | wt | wt | wt | wt | wt | wt | wt | wt | wt |
| **sam21** | wt | wt | wt | wt | 0 | wt | wt | wt | wt | wt | wt | wt | wt | wt | wt+1 | wt |
| **sam22** | wt | wt | wt | wt | wt | wt | wt | wt | wt | wt | wt | wt | wt | wt | wt | wt |
| **sam23** | wt | wt | wt | wt | wt | –1wt | wt | wt | wt | wt | wt | wt | wt | wt | wt | wt |
| **sam24** | wt | wt | wt | wt | wt | –1wt | wt | wt | wt | wt | wt | wt | wt | wt | wt | wt |
| **sam25** | wt | wt | wt | wt | wt | wt | wt | wt | wt | wt | wt | wt | wt | wt | wt | wt |
| **sam26** | wt | wt | wt | wt | wt | wt | wt | wt | wt+2 | wt | wt | wt | wt | wt | wt | wt |
| **sam27** | wt | wt | wt | wt | 0 | wt | wt | wt | wt | wt | wt | wt | wt | wt | –1wt | wt |
| **sam28** | wt | wt | wt | wt | wt | wt | wt | wt | wt | wt | wt | wt | wt | wt | wt | wt |
| **sam29** | wt | wt | wt | wt | wt | wt | wt | wt | wt | wt | wt | wt | wt | wt | wt | wt |
| **sam30** | wt | wt | wt | wt | 0 | wt | wt | wt | wt | wt | wt | wt | wt | wt | wt | wt |
| **sam31** | wt | 0 | wt | wt | wt | wt | wt | wt | wt | wt | wt | wt | wt | wt | wt | wt |
| **sam32** | wt | wt | wt | wt | wt | –1wt | wt | wt | wt | wt | wt | wt | wt | wt | wt | wt |
| **sam33** | 0 | 0 | wt | 0 | 0 | wt | wt | 0 | wt | 0 | 0 | 0 | wt | 0 | wt | wt |
| **sam34** | wt | wt | wt | wt | wt | –1wt | wt | wt | wt | wt | wt | wt | wt | wt | wt | wt |
| **sam35** | wt | wt | wt | wt | wt | wt | wt | wt | wt | wt | wt | wt | wt | wt | wt | wt |
| **sam36** | wt | wt | wt | wt | wt | wt | wt | wt | wt | wt | wt | wt | wt | wt | wt | wt |
| **sam37** | wt | 0 | wt | wt | wt | wt | wt | wt | wt | wt | wt | wt | wt | wt | wt | wt |
| **sam38** | wt | 0 | 0 | wt | wt | wt | wt | wt | wt | wt | 0 | wt | wt | wt | wt | wt |
| **sam39** | wt | wt | wt | wt | wt | wt | wt | wt | wt | wt | wt | wt | wt | wt | wt | wt |
| **sam40** | wt | wt | wt | wt | 0 | –1wt | wt | wt | wt | wt | wt | wt | wt | wt | wt | wt |
| **sam41** | wt | 0 | wt | wt | wt | wt | –1wt | wt | wt | wt | wt | –1wt | wt | wt | wt | wt |
| **sam42** | wt | 0 | wt | wt | wt | wt | wt | wt | wt | wt | wt | wt | wt | wt | –1wt | wt |
| **sam43** | wt | wt | wt | wt | wt | –1wt | wt | wt | wt | wt | wt | wt | wt | wt | wt | wt |
| **sam44** | wt | wt | wt | wt | 0 | wt | wt | wt | wt | wt | wt | wt | wt | wt | wt | wt |
| **sam45** | wt | wt | wt | wt | wt | wt | wt | wt | wt | wt | wt | wt | wt | wt | wt | wt |
| **sam46** | wt | 0 | wt | wt | wt | wt | wt | wt | wt | wt | wt | wt | wt | wt | wt | wt |
| **sam47** | wt | wt | wt | wt | wt | wt | wt | wt | wt | wt | wt | wt | wt | wt | wt | wt |
| **sam48** | wt | 0 | wt | wt | wt | wt | wt | wt | wt | wt+1 | wt | wt | wt | wt | wt | wt |
| **sam49** | wt | 0 | wt | wt | wt | wt | wt | wt | –1wt | wt | 0 | wt | wt | wt | –1wt | wt |
| **sam50** | wt | wt | wt | wt | wt | wt | wt | wt | wt | wt | wt | wt | wt | wt | wt | wt |
| **sam51** | 0 | 0 | 0 | 0 | 0 | –1wt | 0 | 0 | 0 | 0 | 0 | 0 | 0 | 0 | 0 | 0 |
| **sam52** | 0 | 0 | 0 | 0 | 0 | wt | 0 | 0 | 0 | 0 | 0 | 0 | 0 | 0 | 0 | 0 |
| **sam53** | 0 | 0 | 0 | 0 | 0 | –1wt | 0 | 0 | 0 | 0 | 0 | 0 | 0 | 0 | 0 | 0 |
| **sam54** | 0 | 0 | 0 | 0 | 0 | wt | 0 | 0 | 0 | 0 | 0 | 0 | 0 | 0 | 0 | 0 |

# Table 4

| **Genes** | **Cell lines** | **Carcinomas** | **Adenomas*** |
| --- | --- | --- | --- |
| *PPFIA1* | 0% | 0% | - |
| *PPFIA2* | 0% | 0% | - |
| *PTPLA* | 0% | 0% | - |
| *PTPN12* | 0% | 0% | - |
| *PTPN13* | 22.2% | 0% | - |
| *PTPN21* | 26.3% | 16.7% | 11.8% |
| *PTPN23* | 5.3% | 2.0% | - |
| *PTPN4* | 0% | 0% | - |
| *PTPN5* | 0% | 6.0% | - |
| *PTPRA* | 0% | 2.3% | - |
| *PTPRB* | 0% | 0% | - |
| *PTPRE* | 0% | 2.1% | - |
| *PTPRH* | 0% | 0% | - |
| *PTPRR* | 0% | 0% | - |
| *PTPRS* | 10.5% | 12.0% | - |
| *PTPRZ1* | 0% | 0% | - |
